# Early diagnosis of diabetic retinopathy in primary care

**Published:** 2015-03-30

**Authors:** Maria Valeria Jimenez-Baez, Horacio Marquez-Gonzalez, Rodolfo Barcenas-Contreras, Carlos Morales Montoya, Laura Fatima Espinosa-Garcia

**Affiliations:** 1 Coordinación Auxiliar de Investigación en Salud, IMSS Quintana Roo, Cancún, Mexico; 2 Servicio de Cardiopatías Congénitas, Hospital de Cardiología, Centro Médico Nacional Siglo XXI, IMSS, México, Distrito Federal.; 3 Instituto Mexicano del Seguro Social, Hospital Gineco Pediatría No. 07. Cancún Quintana Roo, Mexico; 4 Servicio de Oftalmología, Hospital Regional No 17, Instituto Mexicano del Seguro Social Quintana Roo. Cancún, Mexico; 5 Hospital General de Zona No. 3. Instituto Mexicano del Seguro Social Quintana Roo. Cancún, Mexico; 6 Clinical and epidemiological research group of Instituto Mexicano del Seguro Social in Quintana Roo. Cancún, Mexico

**Keywords:** diabetic retinopathy, diabetes mellitus type 2, diabetic macular edema

## Abstract

**Objective:**

To evaluate the impact of a strategy for early detection of diabetic retinopathy in patients with type 2 diabetes mellitus (DMT2) in Quintana Roo, México.

**Methods:**

Study transversal, observational, prospective, analytical, eight primary care units from Mexican Social Security Institute in the northern delegation of the State of Quintana Roo, Mexico were included. A program for early detection of diabetic retinopathy (DR) in adult 376,169 was designed. Were diagnosed 683 cases of type 2 diabetes, in 105 patients randomized was conducted to direct ophthalmoscopy were subjected to a secondary hospital were assigned. Will determine the degree of diabetic retinopathy and macular edema was performed.

**Results:**

In population were 55.2% female, mean age 48+11.1 years, 23.8 % had some degree of DR, 28.0% with mild non- proliferative diabetic retinopathy 48.0 % moderate 16.0% and severe and 8.0% showed proliferative diabetic retinopathy. Those over age 30 are 2.8 times more risk of developing DR, OR= 2.8; 95%CI: 0.42-18.0, and OR= 1.7; 95%CI: 1.02-2.95 women.

**Conclusions:**

The implementation of programs aimed at the early detection of debilitating conditions such as diabetic retinopathy health impact beneficiaries, effective links between primary care systems and provide second level positive health outcomes for patient diseases.

## Abstract (es)

**Objetivo:**

Evaluar el impacto de una estrategia para la detección temprana de Retinopatía Diabética en pacientes con Diabetes Mellitus tipo 2 (DM2) en Quintana Roo.

**Métodos:**

Estudio Transversal, observacional, prospectivo, analítico. En Ocho unidades de primer nivel de atención de la delegación Norte del Estado de Quintana Roo, México del Instituto Mexicano del Seguro Social. Se diseñó un programa de detección oportuna de retinopatía diabética en 376,169 adultos, se realizó el diagnóstico de 683 casos de DM2, de forma aleatoria se asignaron 105 pacientes a quienes se les practicó oftalmoscopia directa en un hospital de segundo nivel. Se realizó la determinación del grado de retinopatía diabética y edema macular.

**Resultados:**

En la muestra predominaron las mujeres: 55.2%, edad promedio de 48+11.1 años, el 23.8% presentó algún grado de DR, 28.0% con retinopatía diabética no proliferativa leve (DRnPL), 48.0% moderada (DRnPM), 16.0% Severa (DRnPS) y el 8.0% presento Retinopatía diabética proliferativa (DRP). Los mayores de 30 años tuvieron 2.8 veces más riesgo de desarrollar DR, OR 2.8; IC95%: 0.42-18.0 al igual que las mujeres OR= 1.7; IC95%: 1.02-2.95.

**Conclusiones:**

La realización de programas dirigidos a la detección oportuna de enfermedades incapacitantes como la retinopatía diabética tienen impacto en la salud de los derechohabientes, la vinculación efectiva entre los sistemas de atención primaria y segundo nivel ofrecen resultados favorables para la salud de los pacientes.

## Introduction

Diabetes mellitus (DM) is a chronic degenerative disease with a worldwide prevalence of 2-6% 1. According to the World Health Organization, it is estimated that there are currently 150 million people with diabetes and by 2025 this number will double. More than 90% of new cases will be patients with diabetes mellitus type 2 (DM2) 2-4, and in Mexico more than half of these cases are not diagnosed 5. 10% of the general population suffers from DM, which is 3-4% higher than that reported for other countries 6,7.

The main problem with DM is the occurrence of metabolic, vascular and neurological complications 8,9. Diabetic Retinopathy (DR) is one of the most serious DM complications; in most industrialized countries it has become the leading cause of vision loss and blindness among adults 10,11. This condition is of vascular origin, and is characterized by signs of retinal ischemia (microaneurysms, hemorrhages, exudates, intraretinal microvascular abnormalities, abnormalities in the venous caliber and neovascularization) as well as signs of increased vascular permeability 12. This progresses from mild nonproliferative disease, to moderate or severe nonproliferative retinopathy, and finally proliferative disease 10. 

Significant loss of vision results from retinal hemorrhages in the fragile new vessels, with two types of bleeding; that derived from the superficial capillary plexus, which is flame or splinter shaped, and that coming from below, with a mottled or stained appearance; vitreous hemorrhage, macular edema, or retinal capillary hypoperfusion, which leads to scarring and damage to the secondary retina 10. Activation of the local renin-angiotensin system in the eyes of patients with diabetes can directly or indirectly increase growth factor concentration in the vascular endothelium, contributing to angiogenesis and vasopermeability 13. 

Macular edema is caused by abnormal permeability of microcirculation at this level, and is accompanied by hard exudates, which are formed from lipoproteins, soft exudates, myocardial signals in the nerve fiber layer, as well as micro aneurysms and micro hemorrhages; macular edema can occur with any level of retinopathy and is the leading cause of decreased vision 3.

Diabetic Retinopathy development depends on a variety of factors, including time affected by diabetes, effective glucose control, blood pressure and, blood lipid levels. It is more common among Mexico-Americans than non-Hispanic whites; considered as an unexplained risk factor, whereas other sociodemographic factors such as age, medical treatment, education, and gender of the patient are not considered to be risk factors 14.

According to current public health sector policy, patients diagnosed with DM2 require an annual assessment by an ophthalmological specialist, in order to promptly diagnose DR, because otherwise the chance to establish early treatment is delayed; thus the purpose of this study was to demonstrate the impact of a strategy that promotes prompt treatment for patients diagnosed with DM2, by providing immediate ophthalmological attention, in order to determine the degree of DR that exists among patients with newly diagnosed DM2, in units of primary care.

## Materials and Methods

A cross-sectional study was conducted at the Mexican Social Security Institute in Quintana Roo, consisting of eight primary care units (UAP), 4 corresponding to the northern region of the state which were randomly included, comprising a total adult population of 376,169, among whom 683 cases of First Time DM2 were diagnosed over one year, when reporting for the UAP in Cancun, Quintana Roo.

A proportional sample size for a finite population was determined, with a confidence level of 95%, and 80% precision, with an expected ratio of 6.7% for that being evaluated, consisting of 97 subjects in total, with an expected rate of 15% adjustment for loss of sample size, resulting in a final sample that comprised 114 individuals.

**Figure 1.**
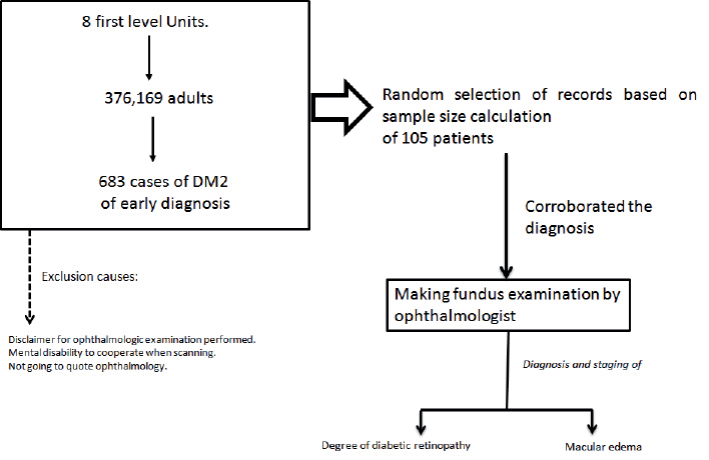
Study General Scheme. Descriptive analytic study of the impact of a program of early detection of Diabetic Retinopathy in patients with DM2 and associated factors.

Firstly patients with first time DM2 diagnosis were identified for each unit assigned No. 13, 14, 15 and 16, as reported by SUAVE (Single System Automated Epidemiological Surveillance), including adults from both genders, and any section of the Unit. Patients who refused to participate in the study, or were proscribed by some mental impairment, were excluded. Once patients were identified, we proceeded to select the sample at random, using a random number table, personally locating affiliates by their phone number registered in the AccDer system (Access to affiliates) IMSS; personal interview was requested with authorization of informed consent and attendance for an appointment with the ophthalmologist for evaluation of the fundus by pupillary dilation, recording the number of cases with diabetic retinopathy and the level of severity for diabetic retinopathy at diagnosis, using the classification for DR produced by the American Association of Ophthalmology (AAO) (Fig. 1).

All patients had their visual acuity measured and a pinhole test was applied, the anterior segment was assessed in search of cataract and rubeosis and an examination of the fundus of the eye was undertaken by pharmacological mydriasis using an indirect ophthalmoscope and lens with three mirrors, among patients where thickening of the macula was identified. All valuations were carried out by the same ophthalmologist retina specialist in order to increase accuracy for diagnosing Proliferative diabetic retinopathy (PDR). Diabetic Retinopathy level was determined according to AAO 3.

This project was registered with the Local Committee for Research and Ethical Research 2301, in strict accordance with the General Health Law in Research Matters, considered as of Minimal Risk, requesting signed informed consent from participants.

Data were analyzed using the statistical program SPSS(r) (version 20.0) for Windows 7. Proportions found in the sample were calculated applying 95% confidence intervals. For the two resulting groups; with and without DR, odds ratio was calculated and a logistic regression model applied.

## Results

The diagnosis of clinically significant macular edema (CSME) was undertaken if any of the following characteristics were present: 1) Thickening within 500 μ of the center of the macula. Exudates within 500 μ from the center of the macula, associated with adjacent thickening. 2) Thickening of a disk or greater area, a disc diameter or less from the center of the macula.

Diabetic Retinopathy classification was carried for each eye of every patient and the most advanced retinopathy identified was taken into account. Those for whom a fundus exploration showed a normal retina were provided with necessary recommendations for annual monitoring of the eye fundus, and patients for whom the presence of some degree of retinopathy was identified were given appropriate treatment. No existing cases of glaucoma were identified.

The total number of registered cases of patients diagnosed with DM2 for the first time was 683, with 114 patients obtained at random, of which nine retired from the study because six emigrated and 3 did not attend the Ophthalmology appointment, leaving a total of 105 patients. 55.2% (58) were women; the mean age was 43±11.1 yrs. Diabetic Retinopathy was found among 25 patients (24.0%) (Table 1). Diabetic Retinopathy was mostly at moderate level of severity (Table 2).

**Table 1.**
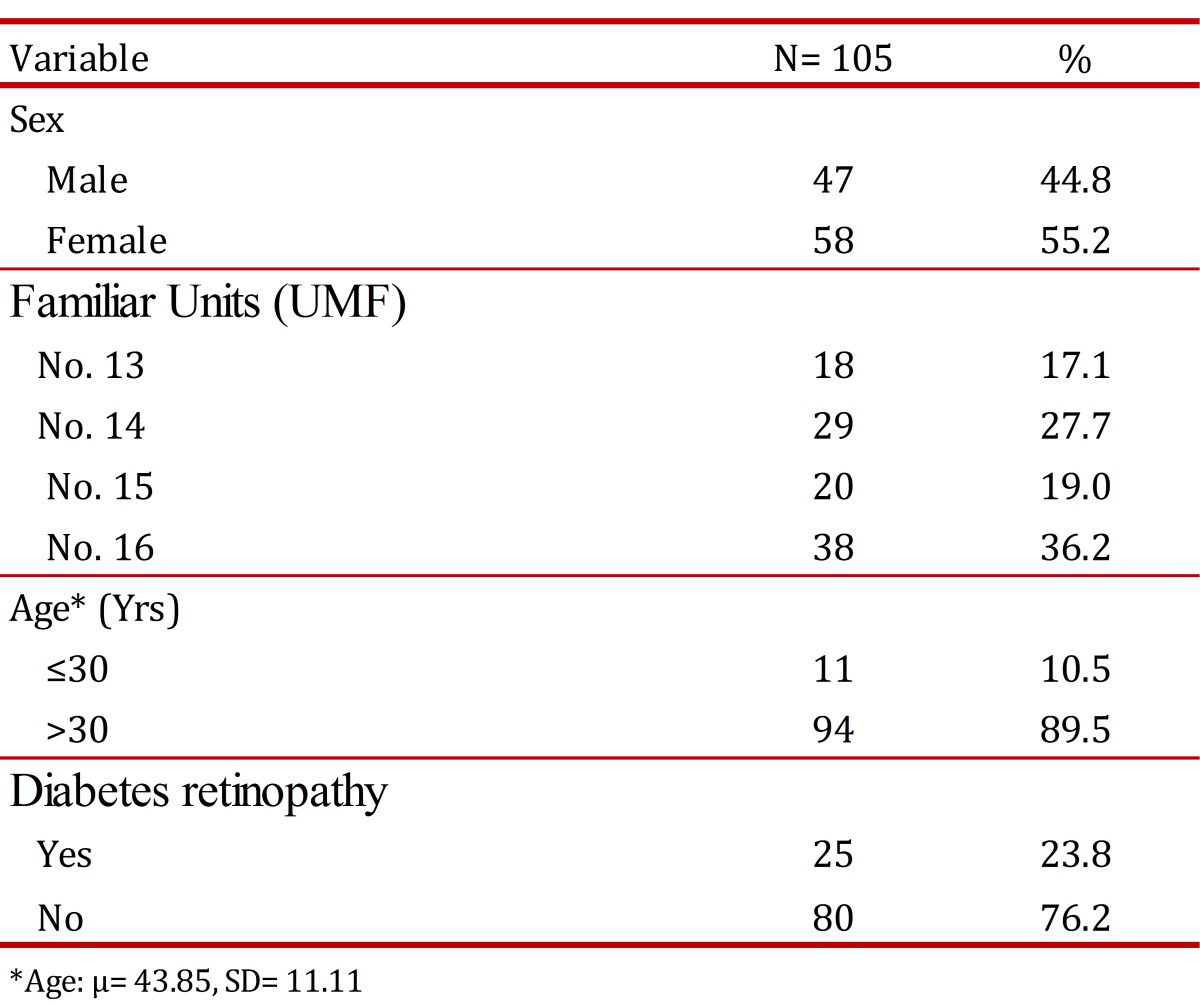
Descriptive data of the population of patients with DM2 with eye examination.

**Table 2.**
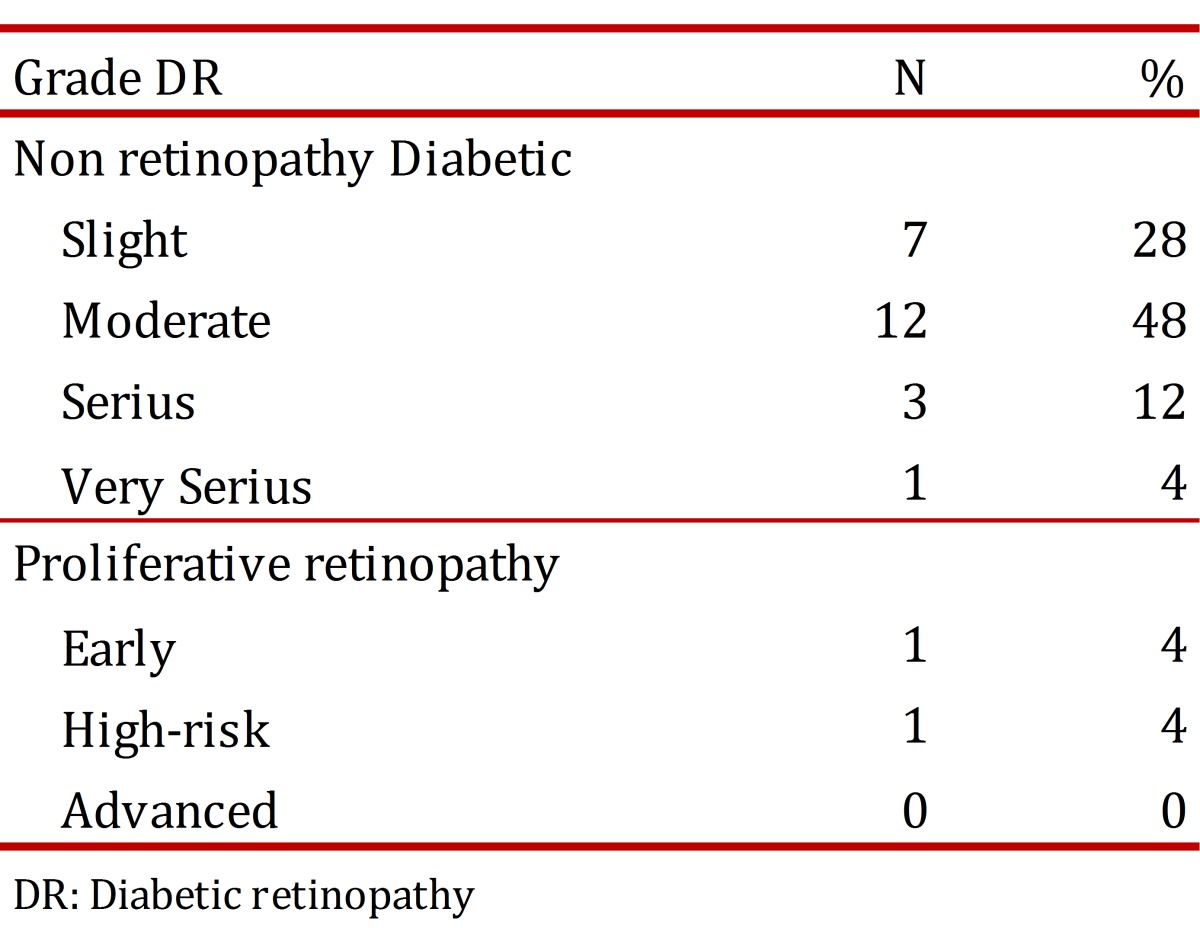
Diabetic Retinopathy Degree.

Risk analysis was conducted to present retinopathy according to age for those younger and older than 30 years, and increased risk was found among patients who were newly diagnosed with type 2 diabetes and who were over 30. Those over the age of 30 are 2.8 times more likely to develop DR, (OR= 2.8; IC95%: 0.42-18.0), as well as women OR= 1.7I; C95%: 1.02-2.95). 

## Discussion

Diabetic retinopathy is a leading cause of blindness among adults, studies in Europe and America suggest the presence of No proliferative diabetic retinopathy (NPDR) and PDR among patients with DM2, with less than 5 years of evolution 9,13. The American Diabetes Association confirmed that 25% of patients who are detected as diabetics may have some degree of DR at the time of diagnosis. The Wisconsin study demonstrates a NPDR of 21% and a PDR of 2%. Similar evidence is available for the Mexican Republic 14, in Durango, Mexico a frequency of 14.5% for NPDR and 1.6% for PDR was obtained among patients diagnosed with DM2 2. In Chiapas, a prevalence of 38.9% for DR was obtained, which was higher than that found in our study 14 ^.^ In the present study, a frequency of 21.9% with NPDR and 1.9% with PDR was found among patients who underwent the diagnosis for DM2, these results being similar to those from reported studies.

Overall, diabetic retinopathy affects 63% of diabetics and increases the risk of blindness 25 times, compared to non-diabetics. 90% of patients with type 1 diabetes and 65% of patients with DM2 develop retinopathy, 10 years after the initiation of the disease 9,14. However, up to 25% of patients newly diagnosed with type 2 diabetes may have retinopathy, at the time of diagnosis 13,14.

The United States reports up to 40 to 45% of patients diagnosed with DM as having some degree of DR, and this is the most common cause of blindness among patients with DM 10,14. This country is where most studies have been conducted on the prevalence of retinopathy and its complications, perhaps due to its high prevalence. Among these, we can mention the Wisconsin Epidemiologic study of Diabetic Retinopathy, where there is prevalence for blindness of 3.6%, among patients with DM1 and 1.6% of those with DM2; among 86% of those with DM1 and a third of those with DM2, DR was secondary 1,9,14. 

The American Diabetes Association (ADA) has found epidemiological evidence indicating that the development of DR initiates at least seven years before type 2 diabetes is diagnosed clinically, so in a patient, who has been newly detected as a diabetic, there may be some degree of DR, but it is essential to identify patients with retinopathy at the time DM diagnosis is made and before their vision is affected, because DR may be present, even if the patient shows no ophthalmologic symptoms; as control needs to be preventive 5. This is the reason why strategies for making prompt assessments of patients diagnosed with DM2 as proposed in this study, are effective and provide better quality of life for patients who are affected, tending to improve control of DM2, a situation which is directly dependent on the patient's habits and the rigor with which they apply their pharmacological and dietary management.

According to various studies, direct ophthalmoscopy, undertaken by ophthalmologists and/or trained technicians, reaches a sensitivity of 80% and a specificity that exceeds 90% and is considered the method of choice as well as being low cost, for diagnosis of diabetic retinopathy and its classification 5,10. 

Intervention, targeting primary care processes as in this case, provides successful results for improving the management of health services, 100% of patients were localized in order to be assessed by ophthalmology.

Direct ophthalmoscopy should be performed by the family physician at the moment that DM2 is diagnosed, however, in this study none of the patients had undergone a previous fundus examination by their family doctor, before being reviewed by the ophthalmology service, likewise, none of them were initially referred by their doctor, all patients denied any symptoms directing them to request an evaluation by an ophthalmologist. 

Clinical practice guidelines have been implemented in primary care for the diagnosis and treatment of diabetes mellitus, which are systematically reviewed by the assigned doctors, however there are hindrances to the implementation of some recommendations such as ophthalmological evaluation, perhaps because of lack of appointments, or sometimes this exploration is performed only among patients with longstanding diabetes, or who have a visual impairment, thus giving more priority to symptoms rather than signs, however on carrying out targeted actions to fulfill these recommendations, benefits are observed for early diagnosis of DR.

 This study is that health service management systems substantially improve, makes clear the need for routine examination of fundus among all newly diabetic patients, which limits damage to retinopathy, when also accompanied by adequate glycemic control.

According to the results obtained after logistic regression analysis, it has been proposed that all women over 30 years with an initial diagnosis of DM2 should receive priority ophthalmological evaluation 14. The age of first diagnosis of DM2 is associated with greater risk of developing diabetic retinopathy, OR= 2.8 and more so if you are female, where the risk increases 3.9 times, in this way and considering that this occurs in developing countries, in health systems that are similar to those in Mexico, the care of these patients can be prioritized, if the epidemiological behavior of this disease is understood.

Weaknesses lie in the mainstreaming of data; monitoring is warranted and even comparisons with other types of problems (renal, sensitive) in order to enrich data and combine monitoring strategies for the control of patients.

It is necessary to demonstrate the need for direct ophthalmology among all diabetic patients and an evaluation by an ophthalmologist physician, providing follow up and complying with that indicated by ADA and the official standard for the control of diabetes mellitus, issued by the Ministry of Health.
